# Milder Phenotype of Homoplasmic Versus Heteroplasmic m.8344A>G Variant in the Same Family: A Case Report

**DOI:** 10.7759/cureus.28490

**Published:** 2022-08-28

**Authors:** Josef Finsterer, Sounira Mehri

**Affiliations:** 1 Neurology, Neurology and Neurophysiology Center, Vienna, AUT; 2 Laboratory of Nutrition and Vascular Health, Faculty of Medicine, Monastir, TUN

**Keywords:** homoplasmy, merrf, myoclonic epilepsy, respiratory chain, mitochondrial

## Abstract

A myoclonic epilepsy with ragged-red fibers (MERRF) patient who carried the m.8344A>G variant in the homoplasmic form manifested a milder phenotype than his sister who carried the same variant in the heteroplasmic form, which has not yet been reported. The 27-year-old male, with an uneventful history, presented at age 19 with fatigue and persistent tremor in both hands. When he talked for a long time, his speech would slow down, and he would stutter. Although electroencephalography showed spike-wave complexes in both occipital projections with generalization, no anti-seizure drugs were given. At age 20, the patient suffered a fall due to muscle weakness. From age 21, generalized myocloni occurred. Because the sister had been diagnosed with MERRF-plus syndrome, the patient underwent genetic testing, which revealed the m.8344A>G variant in homoplasmy. L-carnitine was started. At age 27, the patient experienced a first “syncope” after a long walk, which subsequently recurred up to 2-3 times per day. EEG showed low-amplitude spikes, slow-spike waves at the posterior vertex, and generalized slow-spike waves. Clonazepam was recommended but declined by the patient. In conclusion, the m.8344A>G variant may manifest milder and with a later onset in the homoplasmic as compared to the heteroplasmic form. Further, the homoplasmy of the m.8344A>G variant appears to be more beneficial than harmful.

## Introduction

Myoclonic epilepsy with ragged-red fibers (MERRF) is a rare syndromic mitochondrial disorder that presents as either pure MERRF, characterized by four canonical features - epilepsy, myocloni, ataxia, and myopathy - or as MERRF-plus, which manifests, in addition to the brain, in the peripheral nerves, eyes, ears, heart, gastrointestinal tract, or endocrine organs [1,2]. MERRF is currently understood to be due to 26 mutations in 15 different genes [2]. The most common of these mutations is the m.8344A>G variant in *MT-TK* (tRNA{Lys}), which accounts for 80% of MERRF cases [2]. In the vast majority of cases, the m.8344A>G variant occurs in its heteroplasmic form. Although nearly homoplasmic m.8344A>G variants have occasionally been described [3,4], a homoplasmic patient carrying the m.8344A>G variant is unique, as in the following case.

## Case presentation

The patient is a 27-year-old Caucasian male with uncomplicated early development, who only became conspicuous at age 19 due to fatigue and slight persistent tremor of both hands. When the patient spoke for a long time, his speech would slow down, and he would stutter. Work-up for tremor by electroencephalography (EEG) showed epileptiform discharges in the form of spike-wave activity in both occipital projections with a tendency to generalize in the form of bilateral synchronous discharges. Surprisingly, no treatment with anti-seizure drugs (ASDs) was started at that time. A year later, after a long walk without eating, the patient’s legs buckled and he fell to his knees without losing consciousness. The patient recovered after resting for a week and eating well. After that, he was able to cycle and swim again, but his fatigability persisted. From age 21, the patient suffered recurrent myocloni of the limbs or shoulders. Imaging of the brain did not reveal any meaningful findings. From age 26, impaired coordination was found. At age 27, the patient experienced a first “cerebral blackout” with unconsciousness for a few seconds and stumbling after walking 10 km. Since that episode, he experienced these blackouts 2­-3 times a day. After such an episode, he would occasionally experience discomfort in his head or eyes, or when walking on stones. If such states persisted for a long time, falls occurred, with a loss of consciousness that lasted for a few seconds. The patient needed up to two weeks to recover from such episodes, which subsided spontaneously after one month. At age 27, difficulty concentrating on computer tasks developed. Long periods of working at the computer or reading a book led to fatigue and discomfort, and the patient had to close his eyes.

The family history was positive for MERRF-plus syndrome in the sister [5]. The mother presented with easy fatigability and exercise intolerance at age 30, myocloni from age 52, photosensitivity (i.e., uncomfortable feeling with flickering light and when looking at numerous stones, leaves, or dirty snow on the ground), hypothyroidism, arterial hypertension, and ischemic heart disease.

**Figure 1:**
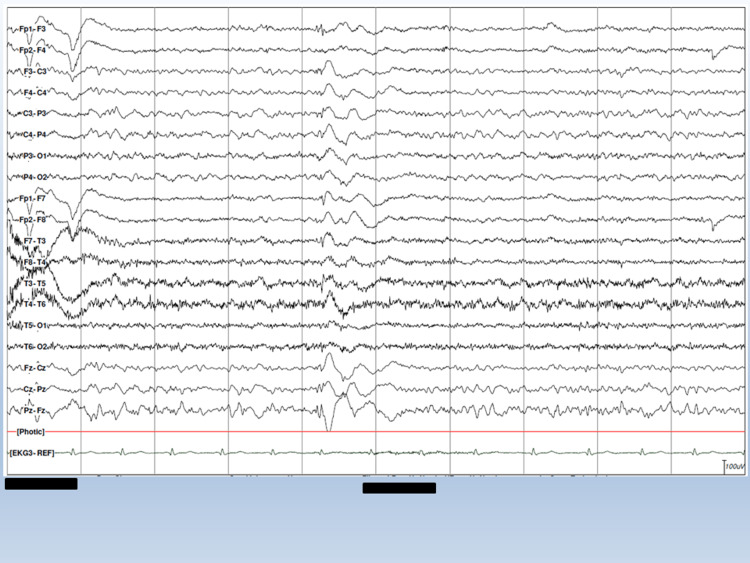
EEG under flickering light at the age of 24 years EEG shows generalized, bilaterally synchronous spike-wave and polyspike-wave complexes

A clinical neurological exam at age 27 showed an impaired convergence response, permanent left eyeball abduction, positional tremor of both hands (more pronounced in the morning than in the evening), occasional myocloni, and static-locomotor and dynamic ataxia. The patient had difficulty standing on one leg, and his tandem gait was impaired. Serum lactate was increased to 3.2 mmol/L (n, 0.00-2.2 mmol/L). EEG revealed low-amplitude spikes, slow spike-wave complexes in the posterior vertex region, and recurrent, generalized slow-spike wave complexes (Figure 1).

Video oculography showed a violation of smooth downward tracking, a violation of vestibuloocular reflex suppression, indicating cerebellar dysfunction, and moderately impaired saccade initiation. Nerve conduction studies revealed mixed axonal and demyelinating sensory neuropathy. Visually evoked potentials were non-informative. Genetic workup for suspected MERRF syndrome, which had been diagnosed in his sister [5], revealed the mitochondrial DNA (mtDNA) variant m.8344A>G in homoplasmy in blood lymphocytes (Table 1). At age 27, the patient started taking L-carnitine. His current medication included L-carnitine (3000 mg/d), vitamin-E (600 mg/d), hydroxypyridine (375 mg/d), coenzyme-Q (300 mg/d), citrulline, and threonyl-lysyl-prolyl-arginyl-prolyl-glycyl-proline diacetate (four drops per day). He refused to take the prescribed ASDs.

**Table 1:** Comparison of genotype and phenotype between the heteroplasmic sister and mother and the homoplasmic index patient +: present, -: absent, mtDNA: Mitochondrial DNA; PCOS: Polycystic ovary syndrome

	Index patient	Sister	Mother
Age (years)	27	34	50
mtDNA variant	m.8344A>G	m.8344A>G	m.8344A>G
mutation load	homoplasmic	heteroplasmic	heteroplasmic
heteroplasmy rate	100%	50%	40%
Onset age (years)	19	7	?
Fatigue	+	+	+
Exercise intolerance	+	+	+
Photosensitivity	+	+	+
Myocloni	+	+	-
Epilepsy	+	+	-
Hypothyroidism	-	-	+
PCOS	-	+	-
Neuropathy	+	+	-
Ataxia	+	+	-

## Discussion

The presented patient was interesting in the following ways: First, the patient had MERRF, which presented with ophthalmoparesis, myocloni, ataxia, tremor, sensory neuropathy, and seizures; Second, MERRF was due to the m.8344A>G variant in homoplasmy, which has not been previously described; and Third, the index patient manifested with a milder phenotype than his sister, who manifested with MERRF-plus and also carried the m.8344A>G variant but with a heteroplasmy of 50% in blood lymphocytes.

The patient’s phenotype met the diagnostic criteria for classical MERRF, but the late onset and the relatively mild phenotype were striking given the homoplasmic distribution of the m.8344A>G variant. Usually, homoplasmy is associated with a more severe phenotype compared to heteroplasmy [6,7], but there is increasing evidence that heteroplasmy often correlates poorly with disease severity [8,9], or that there is no correlation at all [10]. A good correlation between heteroplasmy and severity of the phenotype has been reported especially for MT-ATP6 variants [6]. Homoplasmy of the m.8993T>G variant was associated with severe Leigh syndrome [11], while heteroplasmic variants are associated with less severe neuropathy, ataxia, and retinitis pigmentosa syndrome. In a study of three Chinese families carrying the m.616T>C variant in mt-tRNA(Phe) and uniformly manifesting with isolated chronic kidney disease and hyperuricemia without proteinuria, hematuria, or renal cyst formation, patients carrying the variant in homoplasmy manifested equally to those in heteroplasmy [12].

There are a number of reports of m.8344A>G carriers manifesting with a more severe phenotype when the heteroplasmy rate was high. In a 25-year-old male with exercise intolerance since age 6, stroke-like episodes since age 10, and psychomotor regression and myoclonic seizures since age 12, the m.8344A>G variant, with a heteroplasmy rate of 90%, manifested as mitochondrial encephalomyopathy, lactic acidosis, and stroke-like episodes (MELAS)/MERRF/Leigh overlap syndrome [4]. There are also reports showing that the phenotype can vary between family members carrying the m.8344A>G variant despite a uniform intrapersonal distribution of the mutation load, suggesting that the phenotype is also determined by influences other than the heteroplasmy rate and that the mutation does not change post-natally [13]. For example, a 62-year-old female with MERRF due to the m.8344A>G variant had adult onset despite having a heteroplasmy rate of >90 [14]. 

The reason why the index patient manifested with a milder phenotype than his sister who carried the same variant with a heteroplasmy rate of 50% remains unclear, but it can be speculated that compensatory mechanisms, including anti-oxidative capacity, were better preserved in the index patient than in his sister. It is also conceivable that the mtDNA copy number varied between the two siblings, or that both carried different polymorphisms that modified the phenotype. It can also be speculated that the tissue distribution of homoplasmic variants differs from heteroplasmic m.8344A>G variants, suggesting that homoplasmy prevents broad tissue distribution. Whether the fusion and fission capacity [15] or the capacity of mitophagy differed between the two siblings and thus contributed to the variable phenotypic expression remains speculative. In one large five-generation family, heteroplasmy rates of the m.8344A>G variant correlated with the level of F2-isoprostanes, a specific and reliable marker of oxidative damage, suggesting that the level of oxidative stress contributes to the phenotypic heterogeneity of the variant within a family [16].

## Conclusions

This case demonstrates that the m.8344A>G variant can present with a milder phenotype and with a later onset in the homoplasmic compared to the heteroplasmic form. Contrary to what one would expect, the homoplasmy of the m.8344A>G variant appears beneficial rather than harmful. However, heteroplasmy may not be the only factor determining the phenotypic expression of the m.8344A>G variant.
